# Developmental changes in the extent of drug binding to rat plasma proteins

**DOI:** 10.1038/s41598-023-28434-1

**Published:** 2023-01-23

**Authors:** Fiona Qiu, Katarzyna M. Dziegielewska, Yifan Huang, Mark D. Habgood, Georgia Fitzpatrick, Norman R. Saunders

**Affiliations:** Department of Neuroscience, Central Clinical School, Monash University, Melbourne, VIC Australia

**Keywords:** Blood proteins, Clinical pharmacology

## Abstract

Binding of therapeutics to proteins in blood plasma is important in influencing their distribution as it is their free (unbound) form that is able to cross cellular membranes to enter tissues and exert their actions. The concentration and composition of plasma proteins vary during pregnancy and development, resulting in potential changes to drug protein binding. Here, we describe an ultrafiltration method to investigate the extent of protein binding of six drugs (digoxin, paracetamol, olanzapine, ivacaftor, valproate and lamotrigine) and two water soluble inert markers (sucrose and glycerol) to plasma proteins from pregnant and developing rats. Results showed that the free fraction of most drugs was lower in the non-pregnant adult plasma where protein concentration is the highest. However, plasma of equivalent protein concentration to younger pups obtained by diluting adult plasma did not always exhibit the same extent of drug binding, reinforcing the likelihood that both concentration and composition of proteins in plasma influence drug binding. Comparison between protein binding and brain drug accumulation in vivo revealed a correlation for some drugs, but not others. Results suggests that plasma protein concentration should be considered when using medications in pregnant and paediatric patients to minimise potential for fetal and neonatal drug exposure.

## Introduction

The transfer of compounds, including drugs, from the peripheral circulation into the brain and cerebrospinal fluid (CSF) is limited by specialised cellular barriers, such as the blood–brain barrier, the blood-CSF barrier and the CSF-brain barrier ^1^. Several physical and functional components of these barrier interfaces change during brain maturation, but their passive permeability to lipid insoluble markers remains similar due to tight junctional complexes present from the earliest stages of development ^2^. The passive entry of lipid soluble compounds, on the other hand, can also be controlled at brain barriers by cellular mechanisms such as influx and efflux transporters ^3–5^. In addition to mechanisms specific to these barriers, other factors can also impact the amount of transfer across these interfaces. In blood, many plasma proteins that are responsible for trafficking endogenous substances also interact with circulating therapeutic agents to form complexes that are too large to readily cross cell membranes therefore limiting their tissue distribution ^6^. The extent of protein binding is not only determined by physiochemical properties of the drugs and target proteins and their binding affinity, but also by competition with other substances and the relative concentration of proteins which is known to vary throughout life, during pregnancy and in various states of disease ^7–9^.

Under physiological conditions, the blood brain barrier is largely impermeable to passive transfer of most proteins from blood plasma ^10^. In the CSF, plasma proteins have been shown to be transferred via an intracellular route across choroid plexus epithelium ^11–13^. In the adult their concentration in the CSF is directly related to their molecular size and concentration in plasma ^14^. Drugs that are not bound to plasma proteins are more able to freely penetrate across barrier membranes, such as capillary endothelial cells in the brain. Conversely, it could be expected that highly protein-bound drugs would be retained in the circulation with decreased accumulation of drug in brain and resultant reduction in pharmacological activities at their target sites ^15^. This is often referred to as the *free drug hypothesis* ^15,16^. However, since protein binding is usually a reversible and highly dynamic process, transfer of drugs across brain barriers is also dependent on vascular characteristics, such as flow rate and capillary volume, and rate of dissociation from proteins and membrane permeability ^17,18^. For instance, certain drugs that non-restrictively bind to plasma proteins can more easily dissociate and diffuse across cell membranes ^18^. Nevertheless, the brain and CSF exposure to many substances that are not subject to active transport do correlate with their protein binding ^19,20^.

For some drugs, the ratio of bound to unbound (free) fractions may be sensitive to changes in the concentration of the protein(s) they bind to ^21^. The total concentration of plasma proteins is known to be lower earlier in development in all animal species studied so far ^22–24^. However, although total protein concentration and that of two major drug binding proteins: albumin and ⍺-1-acid glycoprotein, increases with development, the concentration of other plasma proteins, such as ⍺-fetoprotein, fetuin and transthyretin usually decline ^25–29^. It has been suggested that both ⍺-fetoprotein and transthyretin also possess significant drug binding capacities ^30–32^. Physiological variation in plasma protein concentrations also occurs in pregnancy, with the increase in plasma volume resulting in a decrease in albumin concentration ^33,34^.

The degree of drug protein binding is an important factor in understanding their therapeutically active concentrations ^9^. However, potential differences in drug protein binding at different stages of development, due to changes in the concentration and composition of circulating plasma proteins, are often not considered when prescribing medications to pregnant and paediatric populations. In the present paper we have studied this gap in knowledge by investigating in vitro plasma protein binding of 6 drugs (digoxin, paracetamol, olanzapine, ivacaftor, valproate and lamotrigine) and 2 water soluble inert markers of barrier permeability, (sucrose and glycerol) using rat plasma from embryonic (E) day 19 fetuses, postnatal (P) day 4 pups and pregnant and non-pregnant female adult rats. This selection of drugs spans a diverse variety of physiochemical properties such as molecular size, lipid solubility and acidity/basicity. In terms of their therapeutic uses, these drugs consist of both peripherally acting drugs such as digoxin (cardiac glycoside) and ivacaftor (cystic fibrosis transmembrane conductance potentiator) and central nervous system acting drugs such as the antipsychotic drug olanzapine, the analgesic and antipyretic drug paracetamol and the anti-seizure drugs valproate and lamotrigine. The validity of the method used was confirmed using samples from in vivo experiments for two of the drugs (lamotrigine and valproate) that were measured using liquid chromatography coupled to mass spectrometry. In the Discussion the measurements of the drugs’ protein binding have been related to their in vivo brain and CSF levels following systemic administration using previously published data ^35–37^.

## Methods

### Animals

Sprague–Dawley rats used in this study were supplied by the Biological Research Facility at The University of Melbourne. Animals were kept in a 12-h light/dark cycle and were provided with ad libitum access to food and water. Experiments were conducted in pups at embryonic (E) day 19 and postnatal (P) day 4 of both sexes as well as pregnant and non-pregnant female adult rats in accordance with the National Health and Medical Research Committee and the ARRIVE guidelines. All procedures were approved by The University of Melbourne Ethics Committee (Ethics ID: 1714344.1). At all age groups animals came from at least three separate litters.

### Plasma collection & pH adjustment

Time-mated pregnant females at E19 were deeply anaesthetised using intraperitoneal (i.p.) injections of urethane (Sigma-Aldrich) at 2.5 g/kg of body weight and placed on a temperature-controlled heating pad (39 °C). A tracheal catheter was inserted to maintain a clear airway throughout experiment. Individual fetuses were serially exposed via a small incision in the uterine horns. P4 pups and non-pregnant adult females were terminally anaesthetised by inhaled isoflurane (Pharmachem).

Blood samples from E19 and P4 pups, and pregnant and non-pregnant adult females were collected by terminal cardiopuncture from the right ventricle using heparinised syringes or glass micropipettes. Plasma was separated by centrifugation at 1,957xg for 5 min and stored at − 20 °C until use. Because stored plasma samples can become more alkaline after storage, pH was always adjusted back to ~ 7.4 by addition of 1 M hydrochloric acid at 5 μl/ml and confirmed by a blood gas analyser (ABL9 Blood Gas Analyser, Radiometer Pacific) or pH indicator strips (Merck) if sample volume was limited. For each drug, plasma samples from 3 separate adults were used. For E19 & P4 plasma samples from multiple pups were pooled to obtain in most cases 3 individual samples (see Table 3).

### Determination of total protein concentration

Total concentration of proteins in plasma of E19 fetuses, P4 pups, pregnant and non-pregnant adult females was measured using Bradford assay ^38^. Plasma was diluted in phosphate buffered saline at 1:500 for adults, 1:200 for P4 and 1:150 for E19. Ten μl of the diluted plasma samples or protein standards (80 mg/ml HSA and gamma-globulins, Sigma) ranging from 0.031 to 0.5 mg/ml were mixed with 200 μl of Bradford reagent (Bio-Rad, 5000006) in a 96 well plate. Absorbance at a wavelength of 595 nm was measured using a spectrophotometer (Multiskan™ FC Microplate Photometer, Thermo Scientific) and total protein concentration in the plasma samples calculated from the standard curve constructed from the protein standards ^38^.

### In vitro protein binding

Two hundred μl of plasma was spiked with 10 μl of test marker or drug at clinically relevant concentration and traced with 0.1 μCi of their respective radiolabelled form (Table 1). Samples were incubated for 30 min at 37 °C with gentle agitation, intended to be consistent with conditions of in vivo permeability experiments ^35–37^, before undergoing ultrafiltration using 30 kDa molecular weight cut-off centrifugal filters (Centrifree®, Merck Millipore) for 3 min to obtain samples of the protein-free fraction. Filtrate volumes were kept to a minimum of about 15% (< 30 μl) of the total sample volume. Reduction in plasma volume during the process of ultrafiltration has been shown previously to have little effect on binding equilibrium and the concentration of free drugs in the filtrate remained constant up to ~ 40% of the sample volume filtered for some substances^39^. In some experiments adult plasma was diluted to bring total protein concentration in the sample down to levels corresponding to earlier developmental ages ^29^. In these instances, samples were diluted 1:3 or 1:6 in phosphate buffered saline before spiking with compounds as described above. Five ml of scintillation fluid (PerkinElmer Inc.) was added to 10 μl of either whole plasma, which reflects the total plasma drug concentration, or the protein-free plasma filtrate fraction, which only contained unbound drug. Radioactivity in both fractions (disintegrations per minute, DPM) was measured using a liquid scintillation counter (Tri-Carb 4910 TR, PerkinElmer Inc.). Background levels of radioactivity were established using blank plasma samples and these were subtracted from the experimental samples. Results were calculated as radioactivity per μl of sample and free fractions were calculated as a ratio as follows:

$$Free\,fraction \,(fraction\,unbound) = \frac{Protein\,free\,plasma\,DPM/\upmu l}{{Whole\,plasma\,DPM/\upmu l}} \times 100\%$$

**Table 1:** Source, label and concentrations of drugs used.

Compound name	Spiked plasma concentration (μg/ml)	Unlabelled compound source	Radiolabelled form	Radiolabelled compound source
Digoxin (DIG)	0.03	Sigma-Aldrich	[^3^H]	American Radiolabeled Chemicals Inc
Paracetamol (PARA)	15	Sigma-Aldrich	[^3^H]	American Radiolabeled Chemicals Inc
Olanzapine (OLZ)	0.15	Eli Lilly	[^3^H]	American Radiolabeled Chemicals Inc
Ivacaftor (IVA)	40	Selleck Chemicals	[^3^H]	Moravek Inc
Valproate (VPA)	100	Sigma-Aldrich	[^3^H]	Moravek Inc
Lamotrigine (LTG)	20	Sigma-Aldrich	[^3^H]	Moravek Inc
Sucrose	–	–	[^14^C]	Amersham International
Glycerol	–	–	[^14^C]	Amersham International

Concentrations of drugs used were based on previous in vivo studies (digoxin and paracetamol ^35^; ivacaftor ^36^; valproate and lamotrigine ^37^; olanzapine, Huang et al. 2023 in preparation).

### Correlation of protein binding in vitro and in vivo

To validate that the in vitro ultrafiltration method for determination of extent of protein binding reflects protein binding in vivo, two drugs, valproate and lamotrigine, were also tested in vivo in P4 pups. Briefly, animals received an injection of one of the drugs and blood was collected after 30 min reflecting the protocol of the in vitro method and as detailed previously ^35–37^. Immediately following sampling of blood, plasma was separated and used for ultrafiltration as described above. Quantitation of valproate and lamotrigine in whole plasma and the protein-free fractions was performed using liquid chromatography coupled to mass spectrometry (LC–MS/MS) methods established previously ^37^, detailed methods are described in Supplementary Information. Data from in vivo and in vitro measurements are summarised and compared in Table 2. Results obtained using both methods showed a high level of free valproate (80–90%) with a small, but statistically significant difference between the two approaches (*p* = 0.03). The free fraction of lamotrigine, on the other hand ranged between 50 and 60% and was very similar using the two methods (*p* = 0.07, Table 2).

**Table 2:** Comparison of protein binding results from in vitro and in vivo methods.

Drug	In vitro (%)	In vivo (%)
Valproate	92.1 ± 1.3*	78.2 ± 7.7*
Lamotrigine	53.8 ± 4.2	60.2 ± 3.1

Protein-free fraction/whole plasma concentration ratios (%) in plasma of P4 rats from experiments conducted either in vitro (spiked with valproate or lamotrigine with respective [^3^H]-labelled drugs) or in vivo (plasma collected from animals injected with unlabelled drugs estimated by LC–MS/MS). Mean ± SD; n = 3–4. *p < 0.05.

### Statistics

Data analyses were performed using Prism (GraphPad Software Inc.); statistical differences were determined using one-way ANOVA (analysis of variance) with Tukey’s multiple comparisons test between multiple groups or by unpaired Student t tests with F tests between two groups. Groups in which n < 3 were excluded from all statistical analyses. A p value of 0.05 or less was considered statistically significant. Results are presented as mean ± standard deviation (SD) where n ≥ 3.

## Results

### Total protein concentration during rat development and in pregnancy

**Figure 1:**
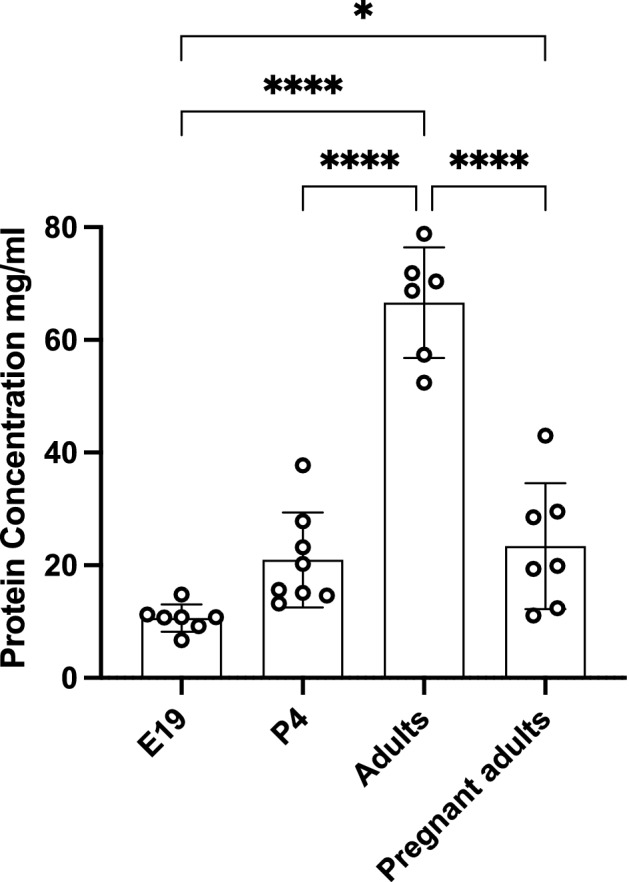
Plasma protein concentration. Total protein concentration in plasma (mg/ml) of E19, P4, non-pregnant and pregnant female adult rats measured using Bradford method. Each point represents an individual animal. Mean ± SD; n = 6–8. One-way ANOVA with Tukey’s multiple comparisons; *p < 0.05, ****p < 0.0001.

Information on total protein concentration in rat plasma throughout development has been published previously ^29^ and is confirmed in the present study for the 4 groups studied: E19 fetuses, P4 pups and in adult pregnant and non-pregnant female rats using the Bradford Assay ^38^. Results are illustrated in Fig. 1. Total protein concentration increased from 10.6 ± 2.4 mg/ml at E19 to 21.0 ± 8.4 mg/ml at P4 and to 66.6 ± 9.8 mg/ml in the adult, making it approximately 3 and 6 times higher compared to P4 & E19 respectively. During pregnancy concentration of proteins decreased significantly to 23.4 ± 11.2 mg/ml (*p* < 0.0001).

### Drug protein binding during development and in pregnancy

Two markers of passive permeability and 6 commonly used therapeutics were used to investigate their plasma protein binding in vitro at E19, P4 and in adults. Results are presented as the ratio of the free fraction (unbound drug) relative to the total plasma concentration as this reflects the proportion of drug immediately available for transfer across the brain and other barriers. All results are summarised in Table 3.

**Table 3:** Free fraction of drugs in plasma of E19, P4, non-pregnant and pregnant female adult rats.

Compound name	E19 (%)	P4 (%)	Non-pregnant adult (%)	Pregnant adult (%)
Digoxin (DIG)	71.3 ± 7.3	81.7* ± 10.6	63.2 ± 3.7	65.1 ± 8.6
Paracetamol (PARA)	87.6 ± 19.7	94.8* ± 2.9	69.4 ± 2.5	84.7* ± 6.2
Olanzapine (OLZ)	64.0 ± 12.7	69.7 ± 7.8	59.8 ± 9.3	68.7 ± 7.3
Ivacaftor (IVA)	0.7	0.7** ± 0.1	0.45 ± 0.04	0.6 ± 0.1
Valproate (VPA)	88.8*** ± 3.1	92.1*** ± 1.3	33.5 ± 6.4	76.2*** ± 5.7
Lamotrigine (LTG)	65.6 ± 7.4	53.8* ± 4.2	69.9 ± 5.0	64.1 ± 8.5
Sucrose	98.6	100.0, 100.0	100.0, 100.0	100.0, 100.0
Glycerol	–	96.9, 100.0	95.9 ± 7.0	95.5 ± 7.8

Protein-free fraction/whole plasma concentration ratios (%) in plasma of E19, P4, non-pregnant and pregnant female adult rats spiked with test compounds and their respective radiolabelled form. Mean ± SD where n = 3; where number of samples were < 3, individual values are listed. Asterisk represents values significantly different to that of non-pregnant adults by unpaired Student t tests with F tests, *p < 0.05, **p < 0.01, ***p < 0.001.

**Figure 2:**
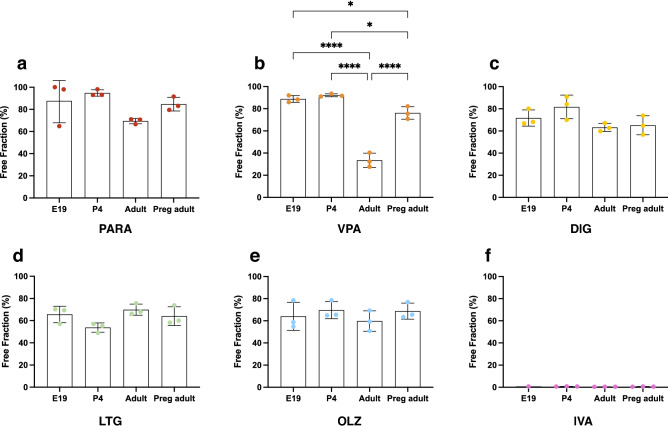
Free fraction of drugs in plasma of E19, P4, non-pregnant and pregnant female adult rats. Protein-free fraction/whole plasma concentration ratios (%) in plasma of E19, P4, non-pregnant and pregnant female adult rats spiked with (**a**) paracetamol (PARA), (**b**) valproate (VPA), (**c**) digoxin (DIG), (**d**) lamotrigine (LTG), (**e**) olanzapine (OLZ), or (**f**) ivacaftor (IVA) with respective [^3^H]-labelled drugs. Mean ± SD where appropriate; n = 1–3. One-way ANOVA with Tukey’s multiple comparisons; *p < 0.05, ****p < 0.0001.

A comparison of plasma protein binding at different ages and in pregnancy by ANOVA is illustrated for each drug in Fig. 2. Only valproate showed statistically significant age-related differences (Fig. 2b) with the free fraction of valproate in adult plasma significantly lower than in E19 fetuses, P4 pups and pregnant adults (*p* < 0.0001). The valproate free fraction in pregnant adults was also significantly lower than E19 fetuses and P4 pups (*p* < 0.05).

Since measurements of drug protein binding are typically made using non-pregnant adult plasma, free-fraction values from E19, P4 and pregnant adult plasma were each compared to non-pregnant adult plasma using student’s t-tests (Table 3). This revealed that free fraction of paracetamol is higher in both P4 and in pregnancy compared to non-pregnant adults (*p* < 0.05). In addition, the free fractions of digoxin and ivacaftor were also higher in P4 than non-pregnant adults (*p* < 0.05 & 0.01), while less free lamotrigine was found in P4 samples (*p* < 0.05, Table 3).

**Figure 3:**
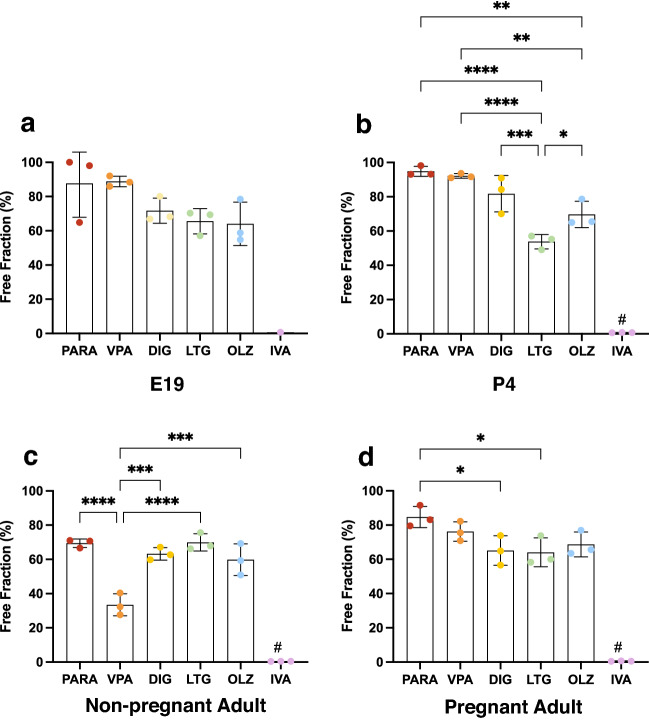
Free fraction of drugs in plasma of E19, P4, non-pregnant and pregnant female adult rats. Protein-free fraction/whole plasma concentration ratios (%) in plasma of E19 (**a**), P4 (**b**), non-pregnant (**c**) and pregnant female adult rats (**d**) spiked with paracetamol (PARA), valproate (VPA), digoxin (DIG), lamotrigine (LTG), olanzapine (OLZ), or ivacaftor (IVA) with respective [^3^H]-labelled drugs. Mean ± SD where appropriate; n = 1–3. One-way ANOVA with Tukey’s multiple comparisons; *p < 0.05, **p < 0.01, ***p < 0.001, ****p < 0.0001. # indicates *p* < 0.0001 with all other groups.

To show potential differences in protein binding between drugs when administered at their clinically relevant doses, comparisons of all tested drugs were made at each age at these appropriate doses scaled to the bodyweight of the animals (Fig. 3). Results showed that ivacaftor was always the most protein-bound (i.e. had the lowest free fraction, < 1%). For the other 5 drugs, there were no significant differences in the extent protein binding in E19 pups (Fig. 3a). However, in P4 animals (Fig. 3b), lamotrigine had second lowest free fraction (53.8 ± 4.2%), followed by olanzapine (69.7 ± 7.8%). In female adult rats, valproate showed extensive protein binding with a free fraction of only 33.5 ± 6.4%, but during pregnancy the free fraction was much higher and similar to other drugs (Fig. 3c,d). Paracetamol appeared to have the highest free fraction in pregnant rats (84.7 ± 6.2%).

### Effects of protein concentration on drug binding

In order to illustrate the effect of developmental increases in plasma protein concentration on drug binding, the free fractions of valproate and lamotrigine in each age group was plotted against the total plasma protein concentration for that group (data from Fig. 1 & Table 3).

**Figure 4:**
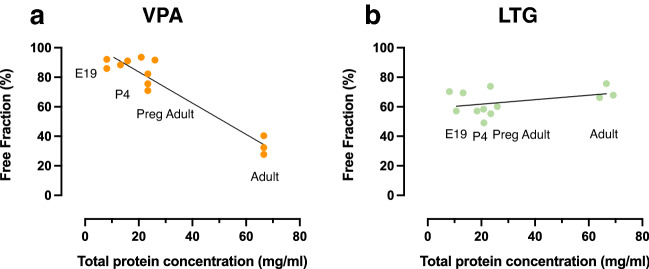
Relationship between free fraction of valproate and lamotrigine and total protein concentration in plasma. Protein-free fraction/whole plasma concentration ratios (%) in plasma of E19, P4, non-pregnant and pregnant female adult rats spiked with valproate (**a**) or lamotrigine (**b**) with respective [^3^H]-labelled drugs plotted against total plasma protein concentration in each group. R^2^ = 0.918 (VPA) & 0.353 (LTG). n = 3.

As can be seen in Fig. 4, the free fraction of valproate appeared to be negatively correlated with total protein concentration in a linear manner (R^2^ = 0.918), whereas the free fraction of lamotrigine appeared to be unaffected by changing concentration of plasma protein.

**Figure 5:**
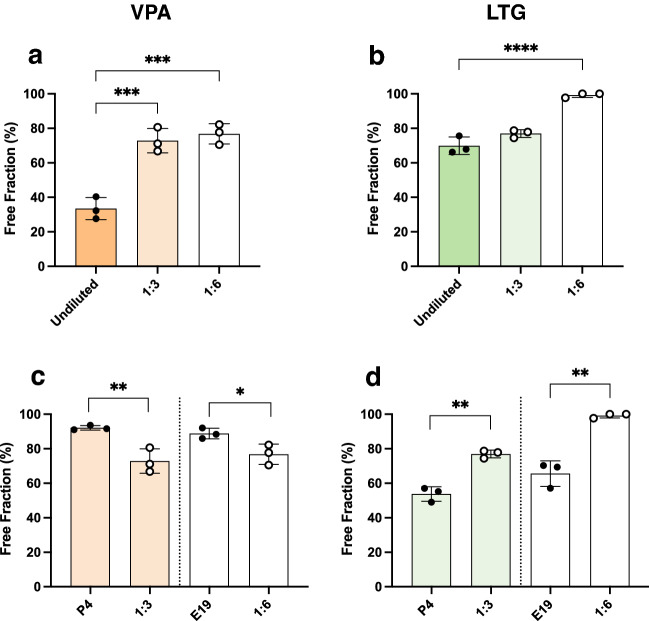
Free fraction of valproate and lamotrigine in diluted adult plasma. Protein-free fraction/whole plasma concentration ratios (%) of valproate (**a**) or lamotrigine (**c**) in undiluted adult plasma compared with adult plasma diluted 1:3 or 1:6 in phosphate buffered saline. (**b**,**d)**. Comparison of free fraction of diluted adult plasma with P4 or E19 plasma of equivalent protein concentration. n = 3. Mean ± SD, n = 3. One-way ANOVA with Tukey’s multiple comparisons test (**a**,**c**) or unpaired Student t tests with F tests between two groups (**b**,**d**); *p < 0.05, **p < 0.01, ***p < 0.001, ****p < 0.0001.

However, during development not only the concentration of protein in plasma changes but the protein composition is also different ^29^. In order to distinguish between effects of protein concentration from the changes in protein composition that are known to occur during rat development, non-pregnant adult plasma was diluted 1:3 and 1:6 to mimic the concentrations in P4 and E19 plasma respectively (Fig. 1 and ^29^). Results for valproate and lamotrigine are illustrated in Fig. 5. Both dilutions resulted in a significant increase in the free fraction of valproate (*p* < 0.001), however there was no significant difference between the 1:3 and 1:6 dilutions. In comparison the free fraction of lamotrigine increased with the 1:6 dilution (*p* < 0.0001), but not at 1:3. When comparing binding of these 2 drugs between diluted adult plasma and plasma from an animal of an age with equivalent total protein concentration (Fig. 5c,d) it was found that in diluted adult plasma there was less free valproate (72.9 ± 7.1%, 92.1 ± 1.3%, 1:3 adult plasma dilution & P4 plasma respectively), but more free lamotrigine (77.0 ± 2.3%, 53.8 ± 4.2%). A similar trend was observed between the 1:6 dilution and E19 plasma. These results indicate that for some drugs total protein concentration alone is not a reliable indicator of their free fraction.

## Discussion

Plasma protein binding of two hydrophilic markers and six commonly used therapeutics was investigated in E19, P4 and pregnant and non-pregnant adult rat plasma in vitro. As anticipated, it was found that the hydrophilic markers sucrose and glycerol showed virtually no plasma protein binding in any of the age groups tested (around 100% free, Table 3) whereas ivacaftor, a highly lipid soluble drug with LogP≈5.8, ^40^, was extensively protein bound in all groups (> 99%). Other drugs covering a broad range of lipid solubility showed different degrees of binding throughout development and in pregnancy. It is generally assumed that plasma protein-bound drug molecules do not exert their CNS therapeutic actions or side effects ^15^ and thus it is important to understand changes in the extent of plasma protein binding in patients at different stages of development and during pregnancy.

Two separate methods used for estimation of the concentration of two of the drugs investigated (valproate and lamotrigine): liquid chromatography coupled to mass spectrometry (LC–MS/MS) and liquid scintillation counting showed a close correlation (Table 2). Additionally, estimation of the extent of plasma protein binding of drugs in vitro was similar to data obtained directly from in vivo experiments. This indicates that our quick and simple in vitro method reflects drug protein binding in vivo for compounds such as lamotrigine. Furthermore, the results from the present study were compared to published data for both the rat and human which is summarised in Table 4. For most drugs there was a reasonable agreement between the protein binding values despite very different methodologies employed. These ranged from spiking plasma in vitro vs using plasma collected from dosed animals/patients, separating free drug fraction by ultrafiltration vs equilibrium dialysis, and several different methods of detection and quantitation of drugs. The two drugs that showed the greatest difference between human and rat data were paracetamol and olanzapine and the drugs that showed good agreement were digoxin, valproate and lamotrigine (Table 4).

**Table 4:** Comparison of free fraction of drugs in plasma of humans and rats.

	Present study (%)	Literature—Rat (%)	Literature—Human (%)
Digoxin	63.2 ± 3.7	59.9 ^65^	75.1 ^65^ 70 ^66^
Paracetamol	69.4 ± 2.5	–	Near 100 ^67^
Olanzapine	59.8 ± 9.3	–	< 7 ^68^
Ivacaftor	0.45 ± 0.04	–	< 3 ^64^ < 1 ^63^
Valproate	33.5 ± 6.4	28 ^69^	10.1 ^42^ 20 ^43^
Lamotrigine	69.9 ± 5.0	60 ^70^	44 ^71^

Protein-free fraction/whole plasma concentration ratios (%) in plasma of non-pregnant female adult rats in the present study and from literature.

**Figure 6:**
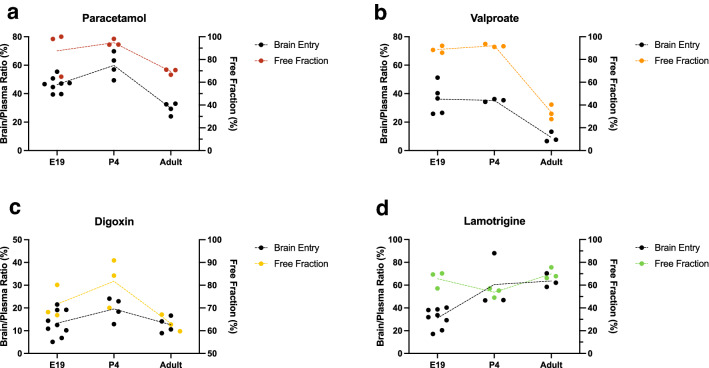
Plasma protein binding and brain entry of drugs in E19, P4 and non-pregnant female adult rats. Protein-free fraction/whole plasma concentration ratios (%) (black, left y-axis) and brain/plasma ratios (%) (colour, right y-axis) of paracetamol (**a**)**,** valproate (**b**)**,** digoxin (**c**) and lamotrigine (**d**). Brain entry results obtained from ^35^ (paracetamol & digoxin), and ^37^ (valproate & lamotrigine).

To examine if there is a correlation between drug binding to plasma proteins and their accumulation in the brain during development in vivo, a comparison between the free fraction obtained in the present study and published information was made. Brain to plasma drug concentration ratios (%) were used as an index of brain accumulation for four of the tested drugs: paracetamol and digoxin ^35^ and valproate and lamotrigine ^37^. In these studies drug accumulation in the brain data were obtained in E19 fetuses, P4 pups and non-pregnant adult rats that were given an i.p. injection of a drug with plasma and brain samples collected 30 min later. This timing is equivalent to the current in vitro protein binding protocol. Comparing changes across development (Fig. 6) reveals that for valproate and paracetamol, both free fraction and brain accumulation were higher in fetal and postnatal pups and decreased substantially in the adults (Fig. 6a,b). This mirrors the known developmental increase in total plasma protein concentration described previously ^29^ and in the present study (Fig. 1). Such a pattern implies that for these two drugs, binding to plasma protein could potentially be a significant factor limiting the amount transferring from blood into brain. Indeed it has been demonstrated in a rat study that the extent of accumulation of valproate in the brain correlates with free drug concentration up to levels at which saturation of plasma protein binding capacity occurs ^41^. In the present study reduced free valproate observed in the adults compared to younger animals might be partially explained by the higher total protein concentration in older animals since plasma protein binding of valproate appears to be directly affected by protein concentration (Fig. 4a). This is in agreement with other studies in both rats and humans ^41–45^. In particular, valproate binding in humans seems to be sensitive to the concentration of albumin, but not ⍺-1-acid glycoprotein ^43,46^. Similarly, data in Fig. 5 also demonstrated that binding of valproate might not be solely dependent on total protein concentration as the protein composition in plasma also changes with development. For example, it is possible that the specific protein(s) responsible for binding of valproate may be present at proportionally higher levels in the adults than in younger animals; therefore, even at a comparable total protein concentration, the extent of binding would be greater in adult plasma, thus limiting its ability to enter into tissues. In contrast, plasma protein binding during development for lamotrigine and digoxin did not correlate with their accumulation in the brain (Fig. 6c,d). The accumulation of lamotrigine in the younger brain was lower than in adults despite its plasma protein binding being similar at both ages. This suggests that other factors likely play a more prominent role in allowing or limiting its transfer across the blood brain barrier. These factors may include the action of influx or efflux transporters on cerebral endothelial cells (Discussed in ^37^).

The implications of variations in free drug concentrations could be of clinical concern especially during pregnancy for women who are reliant on medications to control their conditions ^47,48^. For example, as illustrated in Fig. 2, pregnant rats showed significantly increased levels of free valproate compared to non-pregnant females. Since the total plasma protein concentration decreases in the maternal circulation throughout pregnancy ^49,50^, patients who are routinely administered valproate may experience an increase in free drug levels at the same dose ^51–54^. In addition to reductions in total protein concentration, other factors such as competition with circulating endogenous substances can also affect the extent of valproate binding ^55,56^. It has been observed that changes in free fraction during pregnancy were positively correlated to the level of free fatty acids, but not to other endogenous substances, suggesting that free fatty acids may compete with and displace valproate from its binding sites on plasma proteins ^57^. Similar findings were also observed in a rat study ^58^. Less drug access to protein binding site could also result in elevated exposure of developing fetuses to the drug via the placenta, further contributing to the well-known teratogenic effects of valproate ^59–61^. Additionally, reduced binding to proteins within the fetuses could also allow higher drug entry into the developing brain and exacerbate the known detrimental impacts of valproate on neuro- and cognitive development in drug-exposed fetuses ^62^.

In the present study the number of replicates was limited in some age groups for the inert markers and ivacaftor (n = 1–2), especially in E19 fetuses, since the volume of plasma that can be obtained from each animal is small due to their size. Although statistical analyses could not be performed in those groups, the trend of protein binding over development is supported by results from other age groups and previous work in the literature. For example, ivacaftor appears highly protein bound in P4 and adult animals (< 1% free drug), which accords with other reports ^63,64^, it is therefore likely that it would also have extensive protein binding in E19. Similarly, water soluble markers such as sucrose and glycerol are not expected to bind to plasma protein, which is confirmed by experiments conducted in older rats (Table 1). In addition, each plasma sample represents biological replicates. In cases of pregnant or non-pregnant adults, samples came from different animals. For P4 & E19, each sample was pooled from 3–5 pups/fetuses. Only adult female rats were used in this study as they are compared to pregnant rats for pregnancy-related changes in drug binding.

## Conclusion

Plasma protein binding of some drugs may be a prominent factor in determining their distribution within the body including their transfer across barriers such as the blood–brain barrier and the placenta. Binding of some drugs appears to be dependent on their concentration relative to the total protein concentration and also on plasma protein composition, which changes throughout development and pregnancy. Elevated free drug levels could result in increased fetal and neonatal drug exposure, potentially affecting their sensitive developing brain and leading to long-lasting neuro- and behavioural deficits. Therefore, it would be important to gain a detailed understanding of the protein binding of individual drugs prescribed to certain populations.

## Supplementary Information

Supplementary Information.

## Data Availability

All data generated or analysed during this study are included in this published article.

## Abbreviations

CSF: Cerebrospinal fluid
E: Embryonic day
P: Postnatal day
i.p.: Intraperitoneal
μCi: Microcurie
DPM: Disintegrations per minute
DIG: Digoxin
LC–MS/MS: Liquid chromatography coupled to mass spectrometry
SD: Standard deviation
DIG: Digoxin
PARA: Paracetamol
OLZ: Olanzapine
IVA: Ivacaftor
VAP: Valproate
LTG: Lamotrigine
