# Supplementary material for: Developmental changes in the extent of drug binding to rat plasma proteins
Source: Sci Rep. 2023 Jan 23;13:1266. doi: 10.1038/s41598-023-28434-1 (PMC9870879; doi:10.1038/s41598-023-28434-1)
Supplement: Supplementary file 1 — Supplementary Information. [file 41598_2023_28434_MOESM1_ESM.docx]

**Developmental changes in the extent of drug binding to rat plasma proteins and correlation with their brain entry in vivo**

**Sample preparation for LC-MS/MS**

Methods used for LC-MS/MS analysis in this study are similar to in Toll et al. 2022. Whole and protein-free plasma samples were collected from animals and stored at -20 °C until use. All samples containing lamotrigine were diluted 4-5 times in methanol. Isotope-labelled compounds, valproic acid-d6 (50 μg/ml, Novachem) and lamotrigine-13C, 15N4 (0.7 μg/ml, Supelco), were used as internal standards and were made up immediately before preparation. 10 μl of samples was added to 10 μl of relevant internal standard and made up to 100 μl using methanol. Extraction was performed by mixing samples for 30 seconds with a vortex mixer, followed by centrifuging at 14,000× g for 10 minutes. 50 μl of supernatant was transferred to glass vials for the LC-MS/MS system.

LC-MS/MS analysis was performed using a Vanquish ultrahigh performance liquid chromatography (UHPLC) linked to an Orbitrap Fusion Lumos mass spectrometer (Thermo Fisher Scientific, San Jose, CA, USA) operated at positive ion mode. Mobile phase consisted of 10 mM ammonium formate with 0.1% formic acid in water (Solvent A) and acetonitrile (Solvent B). 5 μL of each sample was injected into an RRHD Eclipse Plus C18 column (2.1×1000 mm, 1.8 μm; Agilent Technologies, Santa Clara, CA, USA) at 50°C at a flow rate of 350 μL/min for 1 min using 5% B. The B% was then increased to 40% in 4 min, 40% to 80% in 0.5 min, kept at 99% for 2 min, decreased to 5% in 0.1 min and maintained for 2.4 min. Peak areas in extracted ion chromatogram (XIC) of monitored product ions from VPA (m/z 143.1016 to 143.1016) and VPA-d6 (m/z 149.1394 to 149.1394) at 6.6 min and LTG-13C, 15N4 (m/z 261.0071 to 212.9830) at 4.4 min were extracted using Skyline 22.2 for quantitative analysis of drugs in each sample.
